# Clinical outcomes of lumbar diseases specific test in patients who undergo endoscopy-assisted tubular surgery with lumbar herniated nucleus pulposus: an analysis using the Japanese Orthopaedic Association Back Pain Evaluation Questionnaire (JOABPEQ)

**DOI:** 10.1007/s00590-019-02574-5

**Published:** 2019-10-08

**Authors:** Jun Komatsu, Masumi Iwabuchi, Tatsuya Endo, Hironari Fukuda, Keigo Kusano, Takuya Miura, Keita Sato, Kazuo Kaneko, Osamu Shirado

**Affiliations:** 1 Department of Orthopaedic and Spinal Surgery, The Aizu Medical Center of Fukushima Medical University, Aizu-Wakamatsu City, Fukushima, 969-3492 Japan; 2 Department of Medicine for Motor Organs, Juntendo University Graduate School of Medicine, Tokyo, 113-8421 Japan

**Keywords:** Endoscopy-assisted tubular surgery, Microendoscopic discectomy, Lumbar herniated nucleus pulposus, Japanese Orthopaedic Association Back Pain Evaluation Questionnaire

## Abstract

**Purpose:**

This study was to evaluate clinical outcomes using a patient-oriented test that scores health-related quality of life (HRQOL) for patients after minimally invasive surgery using microendoscopic discectomy (MED) for lumbar disc hernia. Few studies regarding MED in terms of disease-specific quality of life measures using Japanese Orthopaedic Association Back Pain Evaluation Questionnaire (JOABPEQ) have been published.

**Methods:**

Retrospective analysis of the surgical and clinical outcomes with regard to reducing pain and improving the functional status for 31 patients who underwent MED for lumbar disc hernia was conducted. These patients were evaluated at 3-year follow-up. The evaluations were based on a visual analogue scale (VAS), the Japanese Orthopaedic Association (JOA) scoring system, and the JOABPEQ, which is an objective, patient-oriented test that assesses HRQOL in patients with lumbar disorders.

**Results:**

A low rate of improvement was seen only in mental health until 1 year, the low rate of improvement in mental health and was independently correlated with body mass index (BMI), pre-operative scores on the Brief Scale for Psychiatric problems in Orthopaedic Patients (BS-POP), and scores on the BS-POP at 12 months post-operatively.

**Conclusions:**

All categories of VAS, JOA scores, and all domains of JOABPEQ were significantly higher over 3 years than those obtained pre-operatively. But only mental health domain showed mild improvement until 1 year. Moreover, BMI showed a negative correlation with improvements in the mental health domain post-operatively. As patients may be mentally exhausted from lumbar disc herniation, pre-operative mental health may be improved by surgical treatment.

## Introduction

Reducing muscle damage is important, particularly for the multifidus muscle, which helps maintain partial stability [1]. Minimally invasive decompressive surgical techniques such as microendoscopic discectomy (MED), a technique that uses a tubular retractor and spinal endoscopy for patients with lumbar herniated nucleus pulposus through a unilateral paramedian approach, could lead to better and more aggressive treatment, as well as less muscle and local damage, decreased pain, and faster recovery after surgery [2]. Since being first introduced by Foley and Smith [3], MED has commonly been used to treat lumbar disc herniation. As MED utilizes smaller incisions with the aim of reducing pain and blood loss, it also reduces the duration of hospitalization compared with conventional open laminectomy procedures. Although these advantages have been reported in the literature [4], few studies regarding the superiority of MED in terms of disease-specific quality of life (QOL) measures have been published. Therefore, the purpose of the present study was to evaluate clinical outcomes at more than 3-year follow-up of patients who underwent MED for lumbar disc hernia based on visual analogue scale (VAS) scores, the Japanese Orthopaedic Association (JOA) scoring system (maximum score, 29 points) [5] (Table 1), and the JOA Back Pain Evaluation Questionnaire (JOABPEQ), which is an objective, patient-oriented test that scores health-related QOL (HRQOL) for patients with lumbar disorders [6].

**Table 1:** Criteria of the JOA scoring system

Items	Score
Subjective symptom (9 points)	
Low back pain	
None	3
Occasionally mild	2
Always present or sometimes severe	1
Always severe	0
Leg pain and/or numbness	
None	3
Occasionally mild	2
Always present or sometimes severe	1
Always severe	0
Walking ability	
Normal walking	3
Able to walk 500 m, pain/numbness/weakness present	2
Unable to walk 500 m due to pain/numbness/weakness	1
Unable to walk 100 m due to pain/numbness/weakness	0
Objective finding (6 points)	
Straight leg raising	
Normal	2
30°–70°	1
< 30°	0
Sensory function	
Normal	2
Mild sensory disturbance	1
< Apparent sensory disturbance	0
Motor function	
Normal (MMT normal holds test position against strong pressure or MMT 5)	2
Slightly decreased muscle strength (MMT good holds test position against moderate pressure or MMT 4)	1
Markedly decreased muscle strength (MMT less than fair holds test position (no added pressure) or MMT 3 to 0)	0
Restriction of activities of daily living (14 points)	
Activities of daily living include the following: 7 items (turning over while, lying down, standing, washing one’s face, leaning forward, ability to sit for approximately 1 h, ability to lift or hold heavy objects, and ambulatory ability.)	
None	2
Moderate	1
Severe	0
Bladder function (− 6 points)	
Normal	0
Mild dysuria	− 3
Severe dysuria	− 6
Total	29

## Patients materials and methods

Retrospective analysis of the surgical and clinical outcomes with regard to reducing pain and improving the functional status (QOL) for patients who underwent MED for lumbar disc hernia, performed by a single experienced spine surgeon at a single centre was conducted. Forty-one patients with lumbar herniated nucleus pulposus were treated with MED from June 2015 through May 2016, among whom, 31 (18 males, 13 females; mean age, 51.6 years) with a minimum of 3-year follow-up were evaluated (Table 2). Ten patients were lost to follow-up because they dropped out join to follow-up post-operation, or there were missing entries in the questionnaire. In accordance with the principles of the Helsinki Declaration, all participants provided their written informed consent for participation in the study, as well as for publication of the results and accompanying images. All procedures used in this study were approved by the Ethics Committee of Fukushima Medical University (Approval No. 29263). The indications for surgery were persistent neurological symptoms and failure of conservative treatment over 1 month. Revision cases and patients with mechanical back pain, higher than grade 2 spondylolisthesis, and radiographic findings of instability were excluded. All MED procedures were performed by a single doctor, and post-operative care was standardized for all patients, who were allowed to sit up and walk without lumbar support, walking exercises and muscle training of the lower extremities were started on post-operative day (POD) 1. The drainage tube was removed on POD 2.

**Table 2:** Clinical data for patients who underwent MED from July 2015 to June 2016

Characteristic	Value
Number of patients	31
Follow-up rate (%)	75.6
Age, *n* (years)	51.6
Gender female, rate (%)	45.2
BMI, *n* (Kg/m^2^)	25.1
Operative level	
L2/3	3
L3/4	0
L4/5	10
L5/S	18
Operation time, *n* (min)	84.5
Blood loss, *n* (g)	44.3
Herniation type	
Protrusion	3
Subligamentous extrusion	16
Transligamentous extrusion	4
Sequestration	7
Lateral herniation	1

Both before and after the surgical intervention, the patients’ clinical symptoms and signs of low back pain were evaluated using VAS scores, the JOA scoring system (regarding back pain and pain and numbness in the buttocks and lower limbs), and the JOABPEQ. QOL was evaluated using JOABPEQ of a lumbar disease-specific measure. The Brief Scale for Psychiatric problems in Orthopaedic Patients (BS-POP) was also used. Higher scores of BS-POP are thought to indicate a higher probability of associated lower quality of life (QOL) of the psychiatric factors. These items were evaluated pre-operatively, at 3 months, 1 year, and 3 years post-operatively. The VAS scores ranged from 0 to 10, with 0 = no pain or numbness and 10 = the worst pain imaginable or numbness, for assessing current low back pain, buttock–leg pain, and leg numbness. JOA scores were used to evaluate neurological status and clinical results. All patients were examined before and after surgery to assess the neurological findings and examination results. The JOABPEQ is a patient-oriented instrument that measures HRQOL in patients with lumbar disorders. Several studies used the JOABPEQ evaluated in several lumbar diseases [2, 6, 7]. The average of five categories of JOABPEQ in all patients was similarly distributed, and the JOABPEQ has selected items compared with SF-36, so it is easier to evaluate lumbar diseases. Thus, the JOABPEQ could be used as a specific tool for the evaluation of lumbar diseases. Based on the answers to 25 questions, the examiner calculated five functional scores (low back pain, lumbar function, walking ability, social life function, and mental health). In a verification study by Fukui et al., a significant correlation was found between the patients’ self-ratings and acquired points on the JOABPEQ, suggesting that 20 points could be interpreted as a substantial clinical benefit threshold. Currently, a treatment is judged as “effective” for a particular patient if: (1) the patient provides answers to all the questions necessary to calculate a domain score and shows an increase of ≥ 20 points after the treatment, or (2) the functional score after treatment exceeds 90 points, even if the answer for an unanswered question is assumed to be the worst possible choice [6].

## Statistical analysis

Data were presented as the mean and standard deviation (SD). We assumed that three outcomes (for the VAS, JOA, and JOABPEQ) were essential measures for lumbar disc hernia, so we compared the pre-operative scores with those at follow-up. Statistical analysis was performed using the Mann–Whitney U test and a stepwise multiple regression analysis. Values of *P* < 0.05 were considered statistically significant.

## Results

The 3-year follow-up functional outcome analysis was completed for all 31 patients (18 men [54.8%], 13 women [45.2%]; mean age, 51.6 years; age range, 28–81 years) (Table 2). The disc herniations were at the L2–3 level in three patients, L4–5 in ten patients, and L5–S1 in 18 patients, and there was one lateral hernia patient. There were three protrusive, 16 subligamentous extrusion, four transligamentous extrusion, and seven sequestered disc herniations, as well as one lateral herniation. The mean duration of surgery was 84.5 min (range, 51–125 min), and the mean blood loss during surgery was 44.3 mL (range, 1–377 mL) (Table 2).

### VAS scores for low back pain, buttock–leg pain, and leg numbness

In terms of the VAS scores, low back pain was significantly improved (*P* < 0.001) from 3 months post-operatively, and significant differences were observed from 1 to 3 years post-operatively compared with the pre-operative baseline. Buttock–leg pain and numbness were significantly improved from 3 months post-operatively (*P* < 0.001) (Table 3).

**Table 3:** Scores for the VAS, JOA, and JOABPEQ

	Pre-operative	3-month post-operative scores	1-year post-operative scores	3-year post-operative scores
VAS				
Lumbago	5.4 ± 3.1	1.6 ± 1.5**	1.4 ± 1.2**	0.5 ± 0.5**
Buttock–leg pain	7.5 ± 2.4	1.1 ± 1.8**	1.1 ± 1.5**	0.5 ± 0.7**
Numbness	5.7 ± 3.4	1.4 ± 1.6**	0.7 ± 0.9**	0.4 ± 0.5**
JOA score				
Score	10.0 ± 4.6	27.5 ± 1.3*	28.4 ± 1.0*	28.6 ± 0.7*
(Recovery rate)		(92.6%)	(96.8%)	(97.9%)
JOABPEQ (effective rate)				
Low back pain	29.9 ± 27.4	81.5 ± 24.7** (87.1%)	91.7 ± 17.0** (93.5%)	99.1 ± 5.2** (96.8%)
Lumbar function	47.0 ± 30.6	87.1 ± 15.2** (80.1%)	91.5 ± 10.1** (93.5%)	99.5 ± 3.1** (100%)
Walking ability	38.9 ± 27.1	85.8 ± 20.6** (90.3%)	95.7 ± 8.4** (100%)	99.5 ± 1.7** (100%)
Social life function	32.3 ± 22.9	71.0 ± 22.3** (87.1%)	84.1 ± 15.2** (90.3%)	91.2 ± 13.1** (96.8%)
Mental health	50.2 ± 16.8	65.7 ± 13.6** (29.0%)	66.0 ± 18.3** (48.4%)	81.4 ± 14.8** (74.2%)

These items were evaluated pre-operatively, 3 months, 1 year, and 3 years post-operatively. Values are expressed as means ± standard deviations. Statistical analysis was performed using one-way analysis of variance followed by Bonferroni’s multiple comparison test. Data are expressed as mean ± standard deviation

Recovery rate (%) = (Post-operative score − pre-operative score)/(29 − Pre-operative score) × 100

*P = 0.001 and **P < 0.001

### JOA scores

Following MED, mean JOA scores improved significantly, from 10.0 to 25.9 at 1 month, 27.6 at 3 months, 28.4 at 1 year, and 28.6 at 3 years post-operatively (*P* = 0.001). The mean recovery rates were 92.6% at 3 months post-operatively and 96.8% at 1-year follow-up, and 97.9% at 3 years final follow-up (Table 3).

### JOABPEQ

Regarding the JOABPEQ, significant improvements were seen from 3 months to 3 year post-operatively in the scores for low back pain, lumbar function, walking ability, social life function, and mental health compared with the baseline pre-operative scores (*P* < 0.001) (Table 3). In addition, the “effective rates” on the JOABPEQ [6] for low back pain, lumbar function, walking ability, and social life function improved 87.1%, 80.1%, 90.0%, and 87.1%, respectively, at 3 months. Moreover, at 1 year post-operatively, low back pain, lumbar function, walking ability, and social life function improved 93.5%, 93.5%, 100%, and 90.3%, respectively. On the other hand, only mild improvement was seen for mental health (48.3%). Finally, at 3 years after, low back pain, lumbar function, walking ability, social life function, and mental health improved 93.5%, 93.5%, 100%, 90.3%, and 74.2%, respectively.

Therefore, associations within the mental health domain of 3-month and 1-year post-operative scores were examined using a stepwise multiple regression analysis including age, gender, body mass index (BMI), smoking, prevalence periods, hernia level, operation time, bleeding volume, and VAS, and BS-POP. The analysis showed that the low rate of improvement in mental health was independently correlated with age(odds ratio [OR]: 4.17; 95% confidence interval [CI]: 1.71–10.17; *P* = 0.0017), BMI (OR: 0.58; 95% CI: 0.34–0.987; *P* = 0.044), pre-operative BS-POP (OR: 2.03; 95%CI: 1.05–3.92; *P* = 0.034) and BS-POP scores at 12 months post-operatively (OR: 0.49; 95%CI: 0.27–0.84; *P* = 0.015) (Table 4).

**Table 4:** Risk factors for the low rate of improvement in mental health at 3 months and 1 year post-operatively using a stepwise multiple regression analysis

	Odds rates	95%CI	
Age	**4.17**	**1.71–10.15**	***P* = 0.0017**
Gender	4.61	0.47–45.39	*P* = 0.19
BMI	**0.58**	**0.34–0.99**	***P* = 0.044**
Smoking	0.39	0.06–2.55	*P* = 0.33
Prevalence periods	0.10	0.99–1.1	*P* = 0.51
Hernia level			
L2/3	0.43	0.032–5.78	*P* = 0.52
L4/5	0.15	0.005–3.98	*P* = 0.99
L5/S	0.21	0.022–2.13	*P* = 0.18
Operation times	1.02	0.96–1.08	*P* = 0.38
Bleeding volume	0.99	0.98–1.01	*P* = 0.83
Pre-op. lumbar VAS	0.96	0.71–1.28	*P* = 0.79
Pre-op. buttock pain	0.93	0.63–1.38	*P* = 0.74
Pre-op. numbness	0.97	0.74–1.27	*P* = 0.87
Post-op. 3 month lumbar VAS	1.35	0.67–2.82	*P* = 0.38
Post-op. 3 month buttock pain	1.21	0.63–2.32	*P* = 0.56
Post-op. 3 month numbness	1.10	0.61–1.97	*P* = 0.75
Post-op. 12 month lumbar VAS	2.49	0.81–7.63	*P* = 0.11
Post-op. 12 month buttock pain	1.87	0.57–6.07	*P* = 0.29
Post-op. 12 month numbness	1.27	0.43–3.74	*P* = 0.66
Pre-op. BS-POP	**2.03**	**1.05–3.92**	***P* = 0.034**
Post-op. 3 month BS-POP	0.79	0.56–1.11	*P *= 0.18
Post-op. 12 month BS-POP	**0.49**	**0.27–0.84**	***P* = 0.015**

Higher scores of BS-POP are thought to indicate a higher probability of associated lower quality of life (QOL) of the psychiatric factors

Bold font are significance data.

### Complications

No post-operative complications were observed. There were no complications such as dural tear, wound infection, re-herniation, and addition of fusion surgery.

## Discussion

Several types of new surgical methods for patients with lumbar disc hernia have been developed; however, choosing a surgical method has always been controversial for surgeons. MED was introduced over two decades ago, and its strengths and weaknesses have already been reported [4].

Therefore, this study aimed to evaluate the effectiveness of MED based on clinical outcomes using VAS, JOA, and JOABPEQ scores. The results indicated that MED for lumbar decompression may provide better clinical outcomes, except for mental health until 1 year, suggesting that it is a safe and effective surgical technique that can improve disease-specific QOL. Although the number of patients in this study was small, with only 31 cases, our results are similar to those from a previous study involving pre-operative JOABPEQ scores for lumbar disc hernia [7]. Therefore, we thought that evaluating pre-operative patient status was sufficient to investigate QOL in association with lumbar disc hernia. One previous study reported significant improvements in all domains of post-operative HRQOL [8], and another similarly reported that 40 patients with a L4–5 level hernia had significantly lower increases in mental health scores at 6 months post-operatively [9]. In the present study, to investigate why the mental health domain only showed mild improvements until 1 year, we used a stepwise multiple regression analysis; the results showed that the low rate of improvement in mental health was independently correlated with BMI. These cases that showed lower pre-operative mental health improved after the surgery, higher scores of BS-POP are thought to indicate a higher probability of associated lower QOL of the psychiatric factors, so that the mental health domain of JOABPEQ was positively correlated with mental health scores (BS-POP). A previous study showed a similar return to good mental well-being following surgery, although the procedure in that study was microdiscectomy [10]. In the present study, eight cases (six men, two women) showed low (50 points or fewer) mental health scores pre-operatively, with no further improvement (20 points or more) post-operatively. One of the reasons for this may be that the mean mental health score (50.2 points) was higher than the other domains pre-operatively (Table 3). In other words, patients who did not improve more than 20 points may have already had higher pre-operative mental health scores. On the other hand, a negative correlation was observed by BMI. According to a previous study [11] on BMI and hernia, greater body weight and a higher BMI may affect the development of severe lumbar disc hernia more prominently. However, the influence of anthropometric features on the severity of lumbar disc herniation cannot explain the influence on all patients because of different lifestyles and physical habits. Regarding BMI and mental health, adverse associations reported between adiposity and mental health specific to women have been reported; underweight and obese young women have been shown to have poorer mental health, and middle-aged overweight men have been shown to have better mental health [12]. In addition, increased BMI has been reported in several studies to be associated with poor mental health [13–15], whereas no association or a protective role has also been found [16].

The present study found effects of BMI and psychiatric factors. Management of these issues is needed to obtain better surgical results in the domain of mental health. BMI showed a negative correlation with improvements in the mental health domain post-operatively. Moreover, as patients may be mentally exhausted from lumbar disc herniation, pre-operative mental health may be improved by surgical treatment; however, if no improvement is observed post-operatively, more specialized psychiatric examinations should be considered.

This study had several limitations, the most significant being the lack of a control group, and it is important to study patients undergoing tubular surgery with the assistance of endoscopic surgery and compare the outcomes with those of conventional open surgeries. However, minimally invasive techniques offer the main advantage of faster pain relief, so patients do not choose conventional methods; actually, it has become difficult to compare conventional methods and endoscopic surgery. On the other hand, assessing the outcomes of these techniques with a new patient-oriented test (e.g. JOABPEQ) that scores HRQOL for patients during the early post-operative stage is thought to be important. Moreover, this study was a limited number of the cases, but we had considered to have eliminated bias for clinical outcomes as much as possible by a single experienced spine surgeon technique at a single spine medical center.

## Conclusions

All categories of VAS and JOA scores over 3 year of follow-up were significantly higher than those obtained pre-operatively, and improvements were significantly seen in the JOABPEQ scores for low back pain, lumbar function, walking ability, social life function, and mental health. However, only mental health had showed mild improvement until 1 year. In such cases, more specialized psychiatric examinations should be considered. Management of these issues is needed to obtain better surgical results in the domain of mental health. These results suggest that confirming patient backgrounds before surgery leads to more accurate assessments of low back pain, neurological condition, and QOL and better outcomes after minimally invasive decompressive surgery using MED.
